# Thermo-mechanics aspects of isochoric cryopreservation: A new modeling approach and comparison with experimental data

**DOI:** 10.1371/journal.pone.0267852

**Published:** 2022-04-28

**Authors:** Prem K. Solanki, Yoed Rabin

**Affiliations:** 1 Biothermal Technology Laboratory, Department of Mechanical Engineering, Carnegie Mellon University, Pittsburgh, Pennsylvania, United States of America; 2 Sylvatica Biotech. Inc., North Charleston, South Carolina, United States of America; Tongji University, CHINA

## Abstract

A new mathematical model is proposed for the analysis of thermo-mechanics effects during isochoric cryopreservation. In that process, some ice crystallization in a fixed-volume container drives pressure elevation, which may be favorable to the preservation of biological material when it resides in the unfrozen portion of the same container. The proposed model is comprehensive, integrating for the first time concepts from the disparate fields of thermodynamics, heat transfer, fluid mechanics, and solid mechanics. The novelty in this study is in treating the cryopreserved material as having a pseudo-viscoelastic behavior over a very narrow temperature range, without affecting the mechanical behavior of the material in the rest of the domain. This unique approach permits treating the domain as a continuum, while avoiding the need to trace freezing fronts and sperate the analysis to liquid and solid subdomains. Consistent with the continuum approach, the heat transfer problem is solved using the enthalpy approach. The presented analysis focusses on isochoric cooling of pure water between standard atmospheric conditions and the triple point of liquid water, ice Ih, and ice III (-22°C and 207.4 MPa). The proposed model is also applicable to isochoric vitrification, by substituting the pseudo-viscoelastic material model with the real viscosity model of the vitrifying material. Results of this study display good agreement with phase-diagram data at steady state, and with experimental data from the literature. Furthermore, this study provides a venue to discussing experimentation aspects of isochoric cryopreservation. The proposed model is further demonstrated on a 3D problem, while discussing scale considerations, crystallization conditions, and transient effects. Notably, the new model can be used to bridge the gap between limited pressure and temperature measurements during cryopreservation and the analysis of the continuum. Arguably, this study presents the most advanced thermo-mechanics model to solve practical problems relating to isochoric cryopreservation.

## Introduction

Isochoric cryopreservation—the cryopreservation process in a sealed rigid container—has been presented as a promising approach for the preservation of biological materials in low temperatures, with potential benefits to tissue and organ banking [1–3], as well as to the food industry [4,5]. From a thermodynamics perspective, the term *isochoric* refers to an ideal process occurring within constant-volume boundaries and is used interchangeably with *isovolumetric* and *isometric* in the literature. Isochoric cryopreservation benefits from the elevated pressure with the decreasing temperature within the sealed container. This process is driven by the continuous expansion of liquid water below 4˚C [6], and the subsequent dramatic expansion of water upon ordinary ice formation [7]. When this expansion is constrained in a sealed rigid container, the water is effectively compressed, which results in pressure elevation. It is this pressure elevation under isochoric conditions, which limits further ice formation and thereby is considered beneficial to cryopreservation.

**Fig 1:**
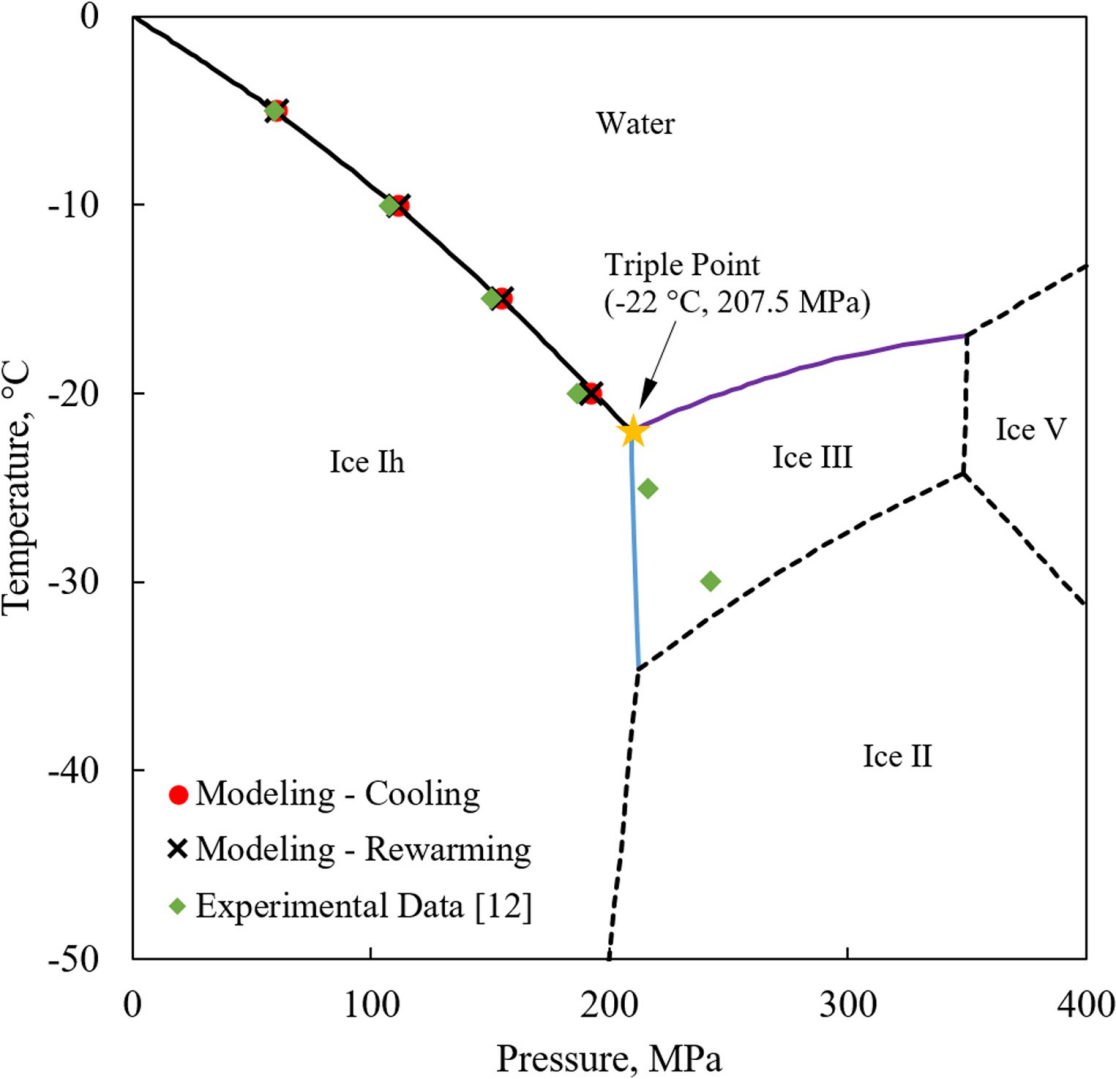
Water phase diagram. Phase diagram of water in temperature and pressure ranges relevant to the current study, as compiled from [11]. Also displayed are experimental results reported in the literature [12] and modeling results developed in the current study for similar conditions.

As with traditional cryopreservation applications, success in isochoric cryopreservation revolves around controlling ice formation, which is the cornerstone of cell and tissue damage [8,9]. Ordinary ice, also known as ice Ih on the pure water phase diagram (Fig 1), is only one of the eighteen known different phases of solid water. The onset temperature of ordinary ice formation itself is intrinsically pressure dependent, which decreases monotonically with the increasing pressure between 0°C at 101.3 kPa (i.e., standard atmospheric conditions) and -22°C at 207.5 MPa (or 2047.8 atmospheres) [10], as displayed in Fig 1. Consequently, cooling water under isochoric conditions within the range of 4°C and -22°C results in a monotonic increase in pressure. Maintaining the cryopreserved material within the unfrozen portion of the isochoric vessel permits storage at lower temperatures without the devastating effects of ice crystallization, and hence the potential benefits to cryopreservation [2,3].

As with other cryopreservation applications, cryoprotective agents (CPAs) can assist in isochoric preservation by modulating ice formation, potentially reducing its hazardous effect. However, while CPAs are primarily designed to suppress crystallization in other applications, some crystallization must occur in the isochoric chamber to drive the potentially beneficial pressure elevation. Furthermore, while the water volume expansion upon cooling within the relevant temperature range is an anomaly, CPAs are expected to contract upon cooling, and the overall pressure elevation in CPA solutions is likely to be lower than that in pure water. In addition, if some pure water crystals form first, the remaining CPA solution will become more concentrated, which would further suppress the crystallization tendency in the remaining CPA solution. Unfortunately, pressure-temperature phase diagrams for CPA solutions are generally not available, where the analysis of such a system is expected to involve significant uncertainties in the material properties.

Despite the above difficulties, isochoric preservation of CPA-loaded tissues has been demonstrated as a viable alternative for non-frozen, short- to midterm storage of tissues and organs [4,13–15]. In general, the preservation of biological materials within the temperature range bounded by the melting point of the CPA solution and the freezing point of pure water ice in standard atmospheric conditions is also known as high-subzero (HSZ) preservation. HSZ isochoric preservation has the potential of reducing the inherent damage due to ice formation, while benefitting from the dramatic decrease of CPA toxicity with the reduced temperature [2]. Nonetheless, pressure-related damage has been reported in tissues and organs stored under prolonged HSZ isochoric conditions [13,16]. Similarly, the benefits of HSZ isochoric preservation of food products have also been demonstrated, using additives such as saline solution, sugars, and some common CPAs, which resulted in improved retention of color, flavor and nutritional values [4,5,17].

While the term *isochoric* refers to the outer boundary condition of the domain, knowledge of the internal amount and distribution of ice is critical to affect cryopreservation success. For example, it is assumed that maintaining the specimen in the liquid space within the isochoric chamber is critical, but the location of ice nucleation is not easy to predict. Furthermore, obtaining detailed experimental observations in isochoric cryopreservation is challenging as the process must be kept behind sealed and rigid walls, where no visualization means or specialized sensors for the task are reported in the literature to the best of our knowledge. Mathematical modeling and computation tools can bridge that gap in knowledge, but an effective modeling approach for isochoric cryopreservation comes with its own challenges. As already alluded above, the thermodynamics properties of pressure and temperature are coupled through geometric constraints in this system, which calls for a system approach to modeling. By contrast, the common cryopreservation modeling approach uses a differential approach, where the phase of state at each unit volume of the domain can be determined independently based on the local conditions. While the latter has been referred to as *isobaric* cryopreservation periodically in the literature of the recent years [17,18], it merely refers to standard surrounding atmospheric pressure despite its implied generality.

Thus far, isochoric cryopreservation modeling has been mostly presented using the underlying principles of thermodynamics [3], while implying that the pressure and temperature are uniformly distributed across the pressure vessel and the process is extremely slow. In thermal sciences terminology, this means an idealized time-independent, quasi-static process. However, the underlying principles of heat transfer teach us that a significant temperature variation should be expected across the isochoric domain, which includes the specimen, CPA solution, and the heavy walls of the container. Furthermore, the underlying principles of solid mechanics suggest that stresses in the solid portion of the domain cannot merely be presented by a single value, such as pressure. In fact, while the temperature is known to be a scalar quantity, meaning that it has only magnitude but no direction, mechanical stress is a tensor, which has nine components at any given point in the domain, characterized by both magnitude and direction.

When an infinitesimal cubic unit volume of a solid is analyzed, six of the mechanical stress components describe shear in orthogonal directions on every two opposing surfaces, while the remaining three components describe normal stress on every two opposing surfaces. In a stationary fluid, as is the case for the unfrozen portion in the isochoric chamber, the shear stresses are practically zero, and the three normal stresses are identical, representing the hydrostatic pressure in a fluid mechanics terminology. However, the authors find no reason to exclude the possibility of different normal and shear stresses to coexist in the solid portion of the domain within the isochoric chamber. This necessitates the incorporation of mechanics concepts in the analysis of isochoric cryopreservation, which relates to both fluids and solids.

To the best of our knowledge, the current study proposes the first thermo-mechanics modeling approach to isochoric cryopreservation. This study presents a computation framework to analyze the coupled problem using commercial FEA codes, with specific examples using COMSOL Multiphysics. The proposed modeling approach is first validated in equilibrium conditions against classical literature data on pure water [11]. The proposed model is then compared with experimental results reported in the literature [12]. Finally, this study is used to provide insight into isochoric cooling and rewarming of pure water in a practical three-dimensional (3D) container.

### Problem definition and mathematical formulation

The mathematical formulation takes the continuum approach, where the solution to the thermo-mechanical problems does not trace the formation of single crystals and their growth, but rather uses the localized average properties of the material. Given the complexity of the bidirectional coupling between the thermal and mechanics modeling, combining temperature-dependent and pressure-dependent material properties, the mathematical formulation is presented in the following order: (i) the geometric model of the problem, (ii) the governing equations of the thermal model, (iii) the physical properties used in the thermal model, (iv) the governing equations of the mechanics model, (v) the physical properties used in the mechanics models, and (vi) aspects of computer modeling.

### Geometric model

**Fig 2:**
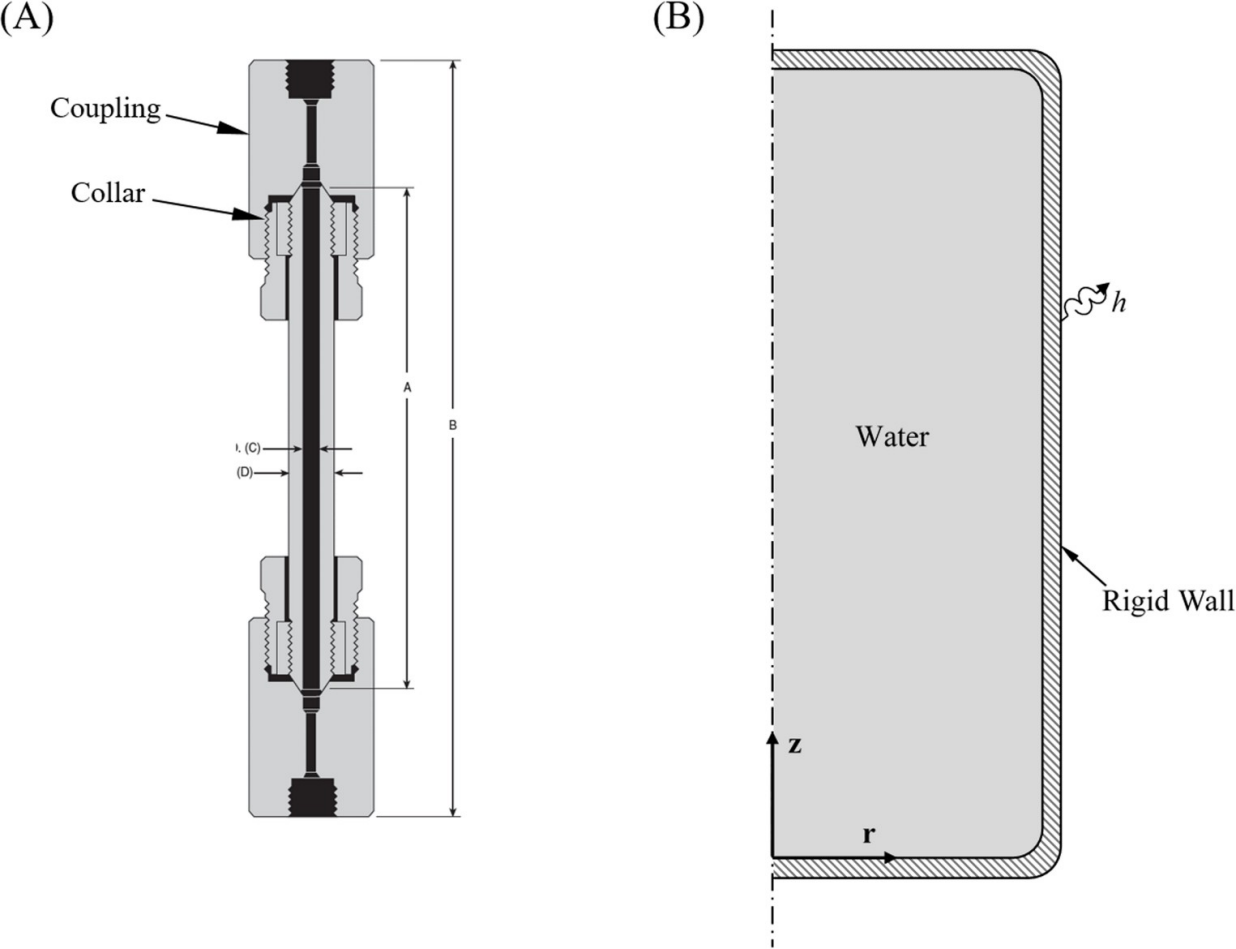
Illustration of the geometric models used in the current study. (A) An MS-1 microreactor (i.e., the isochoric chamber) used in a previous experimental study [12,19] and as a benchmark in the current study. (B) An idealized cylindrical isochoric container used for parametric studies (not drawn to scale).

Two isochoric chambers are analyzed in this study (Fig 2): (A) an isochoric chamber used in a previous experimental investigation [12] as a benchmark, and (B) an idealized cylindrical container of variable dimensions to explore case studies.

The isochoric chamber in the experimental setup, Fig 2A, is a commercially available chamber (MS-1, High Pressure Equipment Company, Erie, PA). This chamber is promoted by that company as a Micro Reactor, but it has a completely passive use in isochoric preservation and the setup should not be confused with a broader class of devices termed microreactors, which are used to facilitate for chemical reactions or energy production. The isochoric chamber used for the experimental study [12] has an internal diameter of 4.76 mm, a length of 177.8 mm, and internal volume of 3 ml. The cylindrical wall is made of Type 316 stainless steel, having a thickness of 4.76 mm. The collar and couplings in Fig 2A are also made of stainless steel.

Due to the stiffness of stainless steel, the thickness of the chamber and the even larger dimensions of the collar and couplings, the experimental apparatus has been approximated as a rigid vessel in previous studies. For the current analysis, the experimental isochoric chamber is approximated as an infinitely long and axisymmetric container for thermal and mechanical analyses, since its internal length is more than 37 times its radius. Therefore, the thermo-mechanical problem in that microreactor can be approximated as a transient and one-dimensional (1D) problem, where the implications of that simplification are discussed below.

The isochoric chamber used for parametric studies is illustrated as a 3D and axisymmetric container in Fig 2B. The rounded edges of the container are included to reduce stress concentration in the mechanics problem, which also allows for a coarser FEA mesh in computer modeling and thereby shorter runtimes. The analysis in this part of the study includes three geometrically similar containers, having a constant ratio of 2.5 between the internal length and internal diameter, in representative volumes of 3 ml, 150 ml and 500 ml, as listed in Table 1. The 3 ml chamber has identical volume to the microreactor used for experiments [12] although in a different length to diameter ratio, the 150 ml was selected as a representative chamber volume for preservation of mammalian cells and rat hearts [13,14], and the 500 ml was selected as a representative chamber volume that could potentially be used for whole organ preservation.

**Table 1:** Thermal response for three representative cases studied in the 3D container shown in Fig 2B, subject to outer surface cooling from an initial temperature of +10°C down to -10°C at a rate of 0.4°C/min, followed by a temperature hold at -10°C thereafter, where *t*_*s*_ is the time in minutes to reach *s* response in percent from the full-scale temperature change (20°C).

Volume, ml	Diameter, mm	Height, mm	*t*_90_	*t*_95_	*t*_97_	*t*_99_
3	11.7	29.2	55	70	96.67	163.3
150	43.1	107.7	92.5	206.7	439.2	1082
500	63.5	158.7	144.2	351.7	813.3	2116

### Thermal model

The thermal model assumes conductive heat transfer throughout the domain:

$$\rho C_{p}\dot{T}=\nabla ∙(k\nabla T)$$ (1)

where *ρ* is the density, *C*_*p*_ is the specific heat, *T* is the temperature, and *k* is the thermal conductivity. Convection heat transfer within the isochoric chamber is neglected due to the relatively small temperature gradients in the system. Heat generation due to mechanical effects are also neglected due to the low strain rate of loading, with more detail provided in the next section.

Convective heat transfer is assumed between the outer surface of the chamber’s wall and the temperature-regulated environment:

$$-k\frac{\partial T}{\partial \hat{n}}=h(T_{s}-T_{\infty })$$ (2)

where $\hat{n}$ is the normal to the wall, *h* is the heat transfer coefficient, and the subscripts *s* and *∞*, represent the outer surface of the wall and the environment, respectively. Radiative heat transfer between the chamber and the environment is small compared to the forced convection in the specific problems analyzed, and therefore is also neglected in this study.

Phase transition modeling is based on the enthalpy approach [20], where a pseudo phase-transition temperature range Δ*T*_*m*_ is assumed for pure water [21]. The enthalpy approach is selected to eliminate the need for freezing front tracing in 3D, which in practice may be represented by one or more curved surfaces. The Δ*T*_*m*_ is selected to facilitate the description of the enthalpy, or the specific heat as its derivative, as a continuous function of the temperature, which tremendously simplifies the computation effort. A practical temperature range of 1 K is selected in this study for Δ*T*_*m*_, while the implications of this selection on the material modeling and results are discussed below. Regardless of that selection, the application of the enthalpy approach with a pseudo phase-transition temperature range is energy conserving [21], which introduces error in temperature calculations only in the vicinity of *T*_*m*_ ± ½ Δ*T*_*m*_ [21].

### Thermal properties

At the core of the material model is the pressure-dependent melting temperature, compiled from phase diagram literature data [11]:

$$T_{m}(P)=-5.701\times 10^{-25}P^{3}+5.892\times 10^{-18}P^{2}-8.363\times 10^{-8}P+273.2[K]$$ (3)

where, *P*, is the gauge pressure (the pressure minus standard atmospheric pressure) given in Pascal. Next, the latent heat is given by [7]:

$$L(T_{m})=1.617\times 10^{-4}T_{m}^{4}-1.711\times 10^{-1}T_{m}^{3}+67.94T_{m}^{2}-1.198\times 10^{4}T_{m}+7.926\times 10^{5}\;[kJ/kg]$$ (4)

where *T*_*m*_ is given in degrees Kelvin.

While the thermal conductivity and specific heat of water are commonly available at standard atmospheric conditions only, the proposed material model assumes that these properties display similar temperature-dependencies in a range of relevant pressures, when the temperature scale is shifted by the melting temperature suppression due to pressure. For this purpose, the adjusted temperature is defined as:

$$T_{adj}(P)=T-T_{sup}(P)\;;\;T_{sup}(P)=T_{m}(P)-T_{m}(P_{0})$$ (5)

where *T*_*sup*_ is the melting temperature suppression between pressure *P* and the standard atmospheric pressure reference *P*_0_. Consistently, the thermal conductivity *k*_*l*_ and specific heat *C*_*p*,*l*_ of liquid water are given by:

$$k_{l}=7.975\times 10^{-9}T_{adj}^{3}-1.583\times 10^{-5}T_{adj}^{2}+8.949\times 10^{-3}T_{adj}-8.691\times 10^{-1}\;[W/m∙K]$$ (6)

$$C_{p,l}=3.625\times 10^{-7}T_{adj}^{4}-5.382\times 10^{-4}T_{adj}^{3}+3.099\times 10^{-1}T_{adj}^{2}-80.41T_{adj}+1.201\times 10^{4}\;[J/kg∙K]$$ (7)

where *T*_*adj*_ is given in Kelvin degrees, and the preadjusted temperature dependencies of those properties are taken from [6]. The thermal properties of ice are given by:

$$k_{s}=9.828e^{-5.7\times 10^{-3}T_{adj}}\;[W/m∙K]$$ (8)

$$C_{p,s}=1.523\times 10^{2}+7.116T_{adj}\;[J/kg∙K]$$ (9)

the preadjusted temperature dependencies of those properties are taken from [22].

A sigmoid function is used to describe the transition of properties from liquid to solid over the phase transition temperature range. In essence, the sigmoid function has the characteristics of a stretched “S”-shaped curve, and it is stretched in such a way that its derivative never changes sign. In practice, there is a family of mathematical expressions which are considered sigmoid functions, where the so-called logistic function is used in the current study to present the modeling approach:

$$f(T)=\frac{1}{1+e^{-a_{0}(T-T_{m}(P))}}$$ (10)

where *a*_0_ is a dimensional coefficient. Since Δ*T*_*m*_ is selected to be 1°C in the current study, a value of 9.19 is selected for *a*_0_. With that selection, the logistics function values are 0.01 and 0.99 at *T*_*m*_ -0.5Δ*T*_*m*_ and *T*_*m*_ +0.5Δ*T*_*m*_, respectively. The logistic function values are practically 0 and 1 for *T*_*m*_ -Δ*T*_*m*_ and *T*_*m*_ +Δ*T*_*m*_, respectively, considering 5 significant digits. It follows that 98% of phase transition occurs within the temperature range of *T*_*m*_ ±0.5Δ*T*_*m*_ and phase transition is practically bounded within the range of *T*_*m*_ ±Δ*T*_*m*_.

Using the logistic function, the thermal conductivity within the phase transition temperature is:

$$k_{m}(T)=f{(T)k}_{l}+[1-f(T)]k_{s}$$ (11)

and, consistently, the specific heat within the phase transition temperature range is:

$$C_{p,m}(T)=f{(T)C}_{p,l}+[1-f(T)]C_{p,s}+L(T_{m})\frac{df}{dT}$$ (12)

where the right-hand side term represents the rate of phase transition.

In practice, due to the logistic function behavior, Eqs (11) and (12) can be used to describe the thermal conductivity and specific heat continually, throughout the domain, without the need to trace the boundaries of the phase transition region.

### Continuum mechanics model

The mechanics model combines the same three water subdomains defined for the thermal model: solid, liquid, and material undergoing phase transition. In order to solve the problem as a continuum rather than a problem having three types of subregions, which may coexist in multiple locations in the domain, the material is modeled as pseudo-viscoelastic. This is an innovative material modeling approach in the current study, which is inspired by viscoelasticity analyses of cryopreservation by vitrification subject to standard atmospheric-pressure surroundings [23–26].

In a common vitrification process, the CPA solution viscosity increases exponentially with the decreasing temperature by more than twelve orders of magnitude, from water-like viscosity at room temperature to such a high value at cryogenic storage that the material can be considered solid in any practical time scale. The temperature threshold below which the material is considered solid is known as the glass-transition temperature. The vitrified material exhibits linear-elastic behavior below the glass transition temperature, just like pure water ice. The innovative approach in the current study approximates water as following a similar viscoelastic behavior, but with a much more rapid elevation of viscosity value over the same phase-transition temperature range from the thermal model, Δ*T*_*m*_. In practice, this rapid elevation in viscosity is about two orders of magnitude faster than that in common cryopreservation by vitrification [27]. It is emphasized that the pseudo viscoelastic effect is included to simplify phase transition tracing only with no practical effect on mechanical stress development, as discussed below.

Consistent with previous mechanics analyses during vitrification, the pseudo-viscoelastic material is modeled as a Maxwell fluid [24,28,29], where the total strain rate is given by:

$${\dot{\varepsilon }}_{total}={\dot{\varepsilon }}_{creep}+{\dot{\varepsilon }}_{elastic}+{\dot{\varepsilon }}_{thermal}$$ (13)

where the creep, elastic and thermal strain rates are calculated by:

$${\dot{\varepsilon }}_{creep}=\frac{S}{2\eta }$$ (14)

$${\dot{\varepsilon }}_{elastic}=\frac{1}{E}[(1+\nu )\dot{\sigma }-\nu I∙tr(\dot{\sigma })]$$ (15)

$${\dot{\varepsilon }}_{thermal}=\frac{1}{3}\frac{\partial e_{v}}{\partial t}I=\frac{1}{3}\frac{\partial }{\partial t}\left(\frac{\rho -\rho_{0}}{\rho }\right)I$$ (16)

where ***σ*** is the stress tensor, ***S*** is the deviatoric stress tensor (that is the difference between the stress tensor and the hydrostatic pressure), *η* is the viscosity, *E* is the elastic modulus, *ν* is the Poisson ratio, ***I*** is the identity matrix, *tr* is the trace of a matrix, *e*_*v*_ is the volumetric strain, and *ρ*_0_ is the initial density upon sealing the isochoric chamber.

Special attention is paid to the thermal strain rate in Eq (16), which is commonly described as the product of the linear thermal expansion coefficient and the temperature rate for isobaric conditions. However, since the problem at hand combines significant pressure elevations due to the isochoric constraints, the thermal strain in the current model also accounts for the compressibility of the material. Assuming small deformations, the linear strain rate in Eq (16) is approximated as one third of the volumetric strain rate, expressed in terms of density. The dependency of density on the linear thermal expansion coefficient and compressibility is described in the Mechanics Properties subsection below.

The pressure is defined as the average normal stress in the solution to the mechanics problem:

$$P=\frac{1}{3}\sum_{1}^{3}\sigma_{ii}$$ (17)

where the three normal stress components in a stationary fluid are identical and equal to the hydrostatic pressure. However, except for special cases, the normal stresses in a solid may vary, which is key to the experimental data interpretation addressed in the discussion section.

Two alternative boundary conditions are considered to model the isochoric system. The first is used to represent the isochoric chamber as an ideal system and thereby eliminating the need for thermo-mechanics modeling in the isochoric chamber walls, while the second alterative is used to model a more realistic system in which the walls may also deform. The ideal system boundary condition assumes infinitely rigid isochoric chamber walls:

$$u(\Omega_{w})=0$$ (18)

where ***u*** is the displacement tensor and *Ω*_*w*_ is its outer boundary of the water domain. The more realistic boundary condition assumes zero normal stress on the outer surface of the isochoric chamber wall, *Ω*_*c*_:

$${\frac{\partial \sigma }{\partial \hat{n}}|}_{\Omega_{c}}=0$$ (19)

Clearly, solution of the problem under the more realistic boundary condition comes at an increased cost of computation.

### Mechanics properties

At the core of the model is the pseudo-viscosity for water, which is defined in the current study as:

$$\eta (T,P)=f(T)\eta_{l}+[1-f(T)]\eta_{s}$$ (20)

with constant values of *η*_*l*_ = 10^4^ Pa·s and *η*_*s*_ = 10^14^ Pa·s for the liquid and solid phases, respectively. The latter value leads the solid phase in the model to behave linear elastically in any practical time scale. As discussed in the context of Eq (10), more than 98% of the change in viscosity occurs over ±0.5°C and it is practically completed over a temperature range of ±1°C (or ±*ΔT*_*m*_).

The density is defined as [7]:

$$\rho (P,T)=\frac{\rho_{r}}{e^{\int_{T_{r}}^{T}\beta (P,T^{\prime })dT^{\prime }-\int_{P_{r}}^{P}\alpha (P^{\prime },T)dP^{\prime }}}$$ (21)

where *β* is the linear thermal expansion coefficient, *α* is the compressibility, the index *r* refers to the reference value, and the prime signifies dummy integration variables. Here, the reference density is selected at standard atmospheric condition: 958.3 kg/m^3^ at the boiling temperature for liquid [6,7], and 916.6 kg/m^3^ at the freezing temperature for solid [7]. For any other combination of temperature and pressure, the density of liquid water can be calculated by integrating Eq (21) along any arbitrary path within the liquid domain of the phase diagram and, consistently, the density of ice can be calculated by integration within the solid domain of the phase diagram. For convenience, these can be stepwise evaluated first by holding the temperature constant and then by holding the pressure constant.

The linear thermal expansion coefficient of liquid and solid water is given by [7,30]:

$$\beta_{l}=(A+\frac{B}{C+D})\left\{ \begin{array}{c} A=a_{lA0}+a_{lA1}T+a_{lA2}T^{2}\\ B=a_{lB0}+a_{lB1}T+a_{lB2}T^{2}+a_{lB3}TD+a_{lB4}D\\ C=a_{lC0}+a_{lC1}T+a_{lC2}T^{2}+a_{lC3}T^{3}\\ D=a_{lD1}P+a_{lD2}P^{2}+a_{lD3}P^{3} \end{array}\right.$$ (22)

$$\beta_{s}=a_{s0}+a_{s1}T+a_{s2}T^{2}+a_{s3}T^{3}$$ (23)

which is calculated in K^-1^, *T* is the temperature in degrees Kelvin, *P* is the pressure in Pa, and the coefficients for Eqs (22) and (23) are listed in Table 2.

**Table 2:** Coefficients for thermal expansion of water and ice, Eqs (22) and (23).

	Coefficient	Value	Coefficient	Value
Water, Eq (22)	*a*_*lA*0_	4.785×10^1^	*a*_*lC*0_	-4.281×10^3^
	*a*_*lA*1_	-8.128×10^−2^	*a*_*lC*1_	-3.391×10^1^
	*a*_*lA*2_	8.498×10^−5^	*a*_*lC*2_	3.658×10^−1^
	*a*_*lB*0_	5.560×10^5^	*a*_*lC*3_	-5.896×10^−4^
	*a*_*lB*1_	-3.763×10^3^	*a*_*lD*1_	1.0×10^−5^
	*a*_*lB*2_	5.563	*a*_*lD*2_	3.289×10^−14^
	*a*_*lB*3_	5.596×10^−3^	*a*_*lD*3_	-2.659×10^−23^
	*a*_*lB*4_	-2.765×10^1^		
Ice, Eq (23)	*a*_*s*0_	-1.250×10^−2^	*a*_*s*2_	-5.561×10^−7^
	*a*_*s*1_	1.452×10^−4^	*a*_*s*3_	7.110×10^−10^

The compressibility of liquid and solid water is given by [7,30]:

$$\alpha_{l}=8.860\times 10^{-46}P^{4}-2.081\times 10^{-36}P^{3}+1.998\times 10^{-27}P^{2}-1.092\times 10^{-18}P+4.417\times 10^{-10}\;[{Pa}^{-1}]$$ (24)

$$\alpha_{s}={\left(5\times 10^{-10}P-1.199\times 10^{-3}T+1.173\right)}^{-1}\;[{Pa}^{-1}]$$ (25)

where the temperature is given in degrees Kelvin and the pressure in Pa.

Similarly to the thermal properties, the density is continually defined as:

$$\rho (T,P)=f(T)\rho_{l}+[1-f(T)]\rho_{s}$$ (26)

Finally, a constant elastic modulus of 10 GPa and a constant Poisson’s ratio of 0.33 are assumed temperature- and pressure-independent throughout the domain [31].

For the more realistic cases where the container walls are assumed to be strained by the elevated pressure in the isochoric chamber as reflected from Eq (19), stainless-steel Type 316 wall material properties are assumed (Table 3), consistent with the benchmark experimental study. In this case, the steel is considered as linear-elastic.

**Table 3:** Material properties of stainless-steel Type 316 used for modelling the isochoric chamber walls.

Property	Value	
Density, *ρ* (kg/m^3^)	$8.058\times 10^{3}-1.964\times 10^{-1}T-4.831\times 10^{-4}T^{2}+4.114\times 10^{-7}T^{3}-1.338\times 10^{-10}T^{4}$	[32,33]
Thermal conductivity, *k* (W/m·K)	$7.956+2.084\times 10^{-2}T-4.707\times 10^{-6}T^{2}+6.271\times 10^{-10}T^{3}-1.241\times 10^{-12}T^{4}$	[34]
Specific heat, *C*_*p*_ (J/kg·K)	$-75.58+5.007T-1.649\times 10^{-2}T^{2}+2.027\times 10^{-5}T^{3}$	[34]
Elastic modulus, *E* (Pa)	$2.059\times 10^{11}+9.297\times 10^{7}T-1.091\times 10^{6}T^{2}+3.231\times 10^{3}T^{3}-3.652T^{4}$	[35,36]
Poisson’s ratio, *ν*	0.29	[35,36]
Thermal expansion coefficient, *β* (1/K)	$1.102\times 10^{-5}+3.204\times 10^{-8}T-6.456\times 10^{-11}T^{2}+5.046\times 10^{-14}T^{3}$	[32]

### Computer modeling

The thermo-mechanics problem in this study is solved using the commercial FEA software COMSOL Multiphysics^®^ [37]. The coupling between the thermal and mechanical problems is brought about through the material properties, which are temperature-dependent (calculated by heat transfer solver), pressure-dependent (calculated by solid mechanics solver), and through the coupling physical effect of thermal strain. The axisymmetric domain is discretized into triangular elements based on a mesh convergence study (ranging between 6000 to 15000 elements, depending on the system volume), using an inbuilt meshing module in COMSOL Multiphysics^®^ [38]. Linear elements are used to solve the heat transfer problem, while bi-quadratic elements are used to solve the mechanical problem.

**Fig 3:**
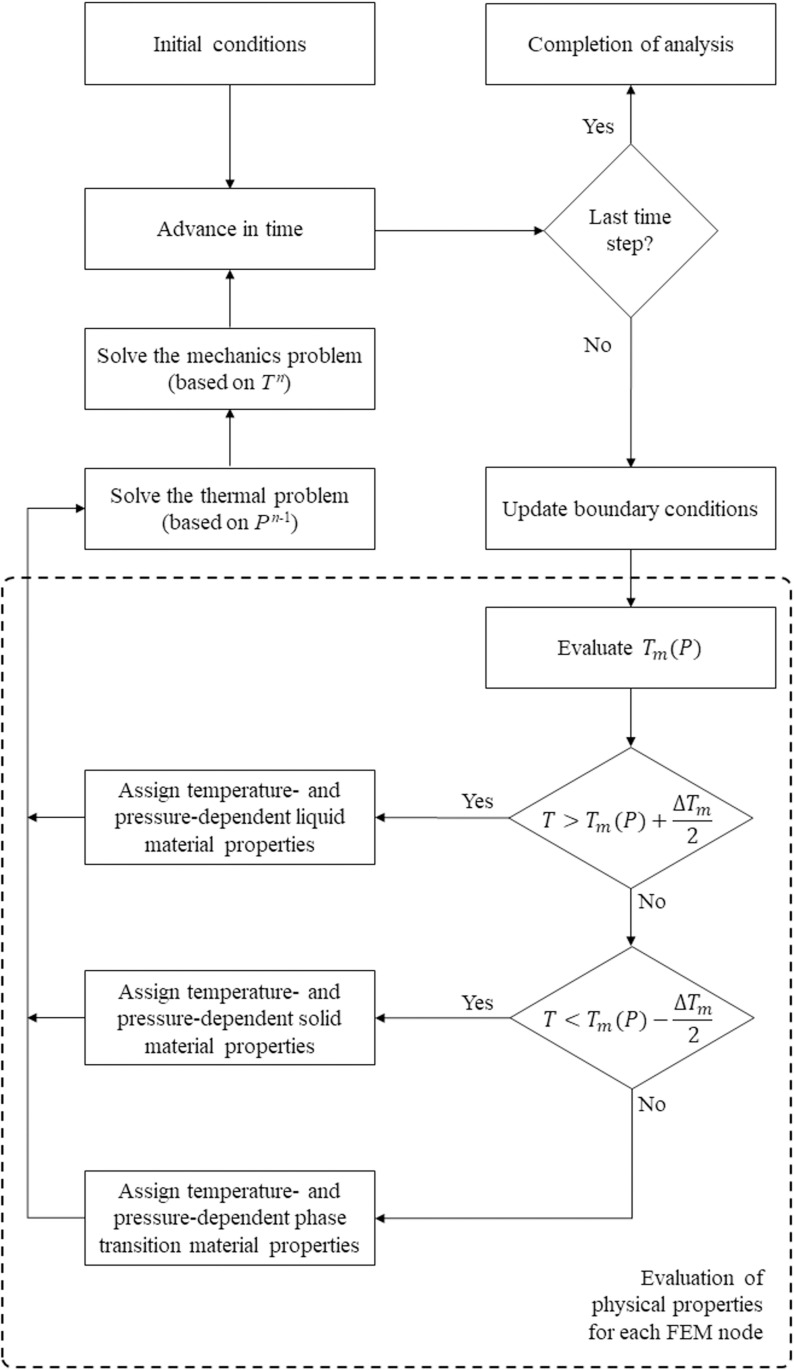
Flow diagram for computation using the proposed model. The symbol *n* is a time index.

The computation process is illustrated in Fig 3. At the core of the computation process is the identification of the melting temperature distribution based on the localized pressure at every point in the domain. Next, the temperature-dependent and pressure-dependent properties are evaluated at each grid point in the domain, based on the adjusted temperature. Subsequently, the thermal problem is solved first based on the pressure distribution in the previous time level, *n*-1, while the mechanics problem is solved second based on the temperature at the current time level *n*. This numerical approximation by incremental decoupling of the thermal and mechanical problems is incorporated to avoid circular dependency on the pressure in the computation process. The effect of this approximation is minimized by limiting the maximum time step size to 2.5 s, based on a time step convergence study. Finally, the boundary conditions are updated at the end of each time step in preparation for a new cycle of solution. This process progresses until it reaches a predetermined time limit.

**Fig 4:**
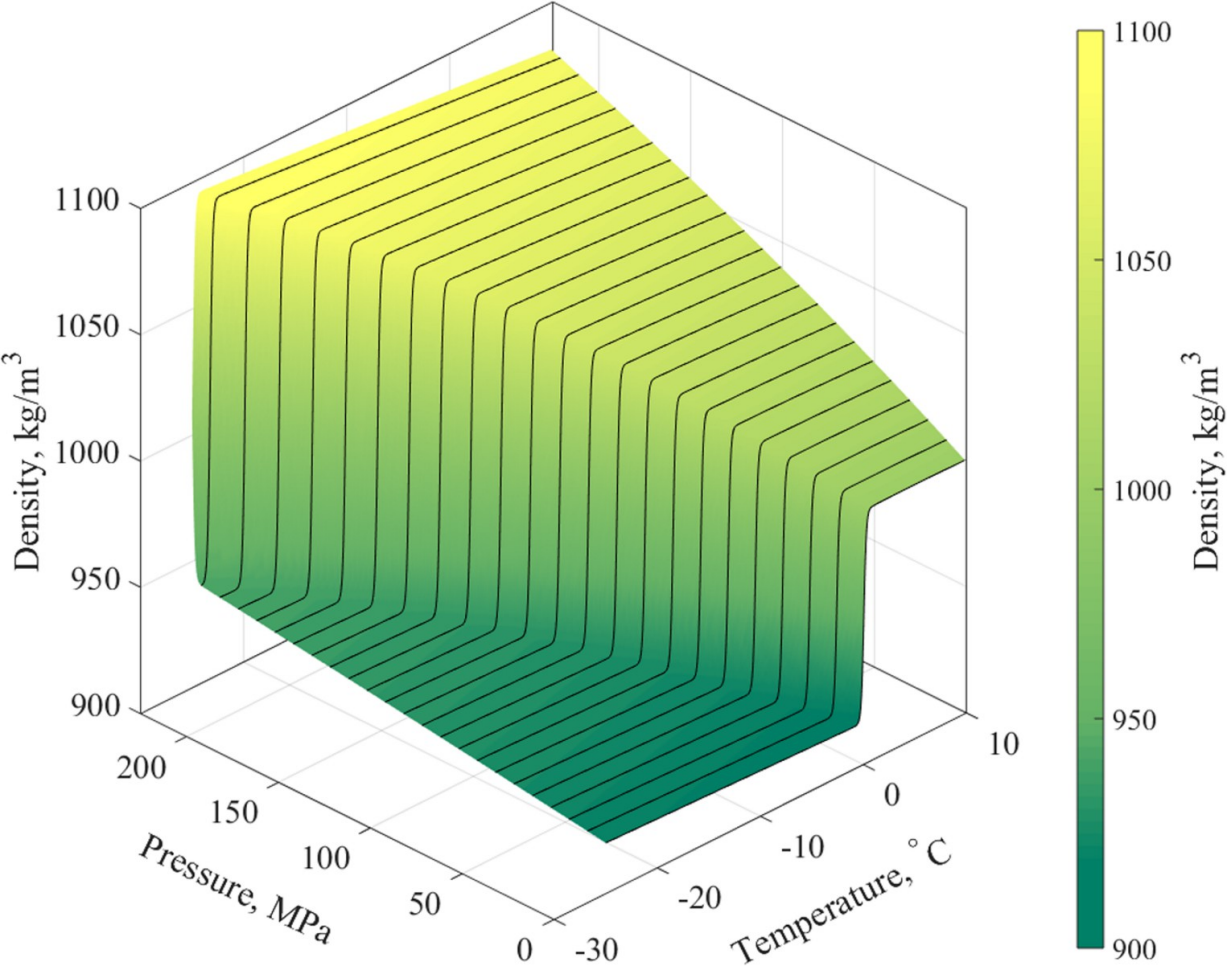
Water density. Density of water within the range of pressures and temperatures investigated in the current study, as calculated by Eq (26).

A typical runtime for the problems presented in this study is in the range of 8 to 24 hours, using an Intel Core i7-6700 machine having 16 GB of RAM. In order to accelerate computation runtime, a search table for the density has been prepared a priori based on Eqs (21)-(26), as depicted in Fig 4.

## Results and discussions

The discussion in this study is divided into two sections: (i) validation of the model against literature data, and (ii) gaining insight into scale-up isochoric cryopreservation. Reference literature data used for the first section combine two sources: the widely available pressure-temperature phase diagram of water [11], and experimental data obtained recently by Ukpai et al. [12]. From the outset, the authors very much appreciate the opportunity to compare the proposed modeling results with data obtained by an experimental setup relevant to concurrent efforts in cryopreservation, which presents a unique opportunity to not only discuss modeling aspects, but also experimental considerations and data interpretation.

### Benchmarking with experimental data

Model evaluation with experimental data has been performed on the benchmark isochoric chamber illustrated in Fig 2A following the experimental protocol described in [12]. The isochoric chamber is assumed to have rigid walls for this comparison, as described by Eq (18). The experiment was conducted by immersing the isochoric chamber in a water-ethylene glycol bath of a commercial cooling system (NesLab RT-140, Thermo Fisher Scientific, Waltham, MA, USA). There, the controlled variable is the temperature of the alcohol solution cooling bath, rather than the temperature inside the experimental isochoric chamber.

**Fig 5:**
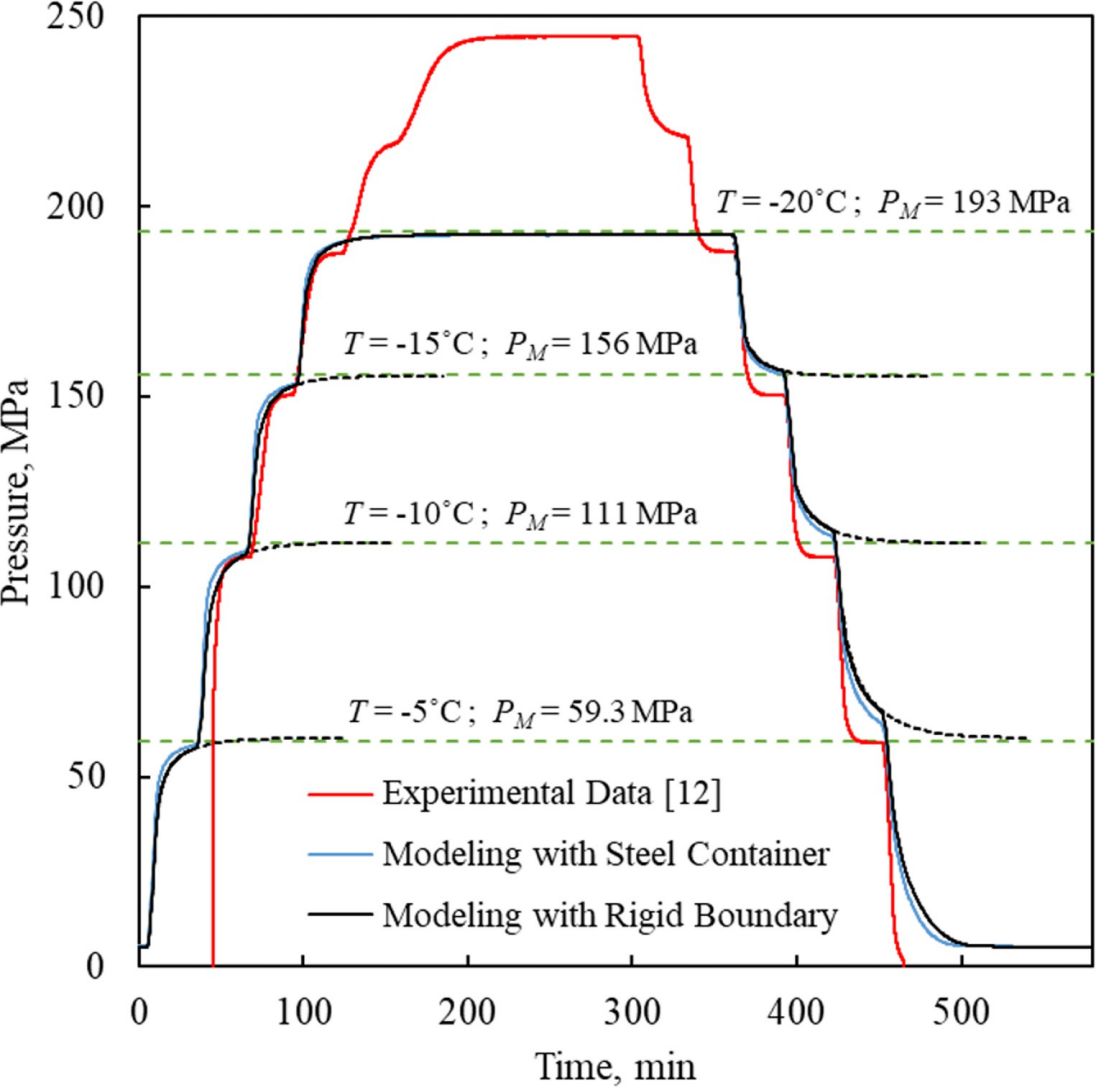
Comparison of experimental data [12] and 1D modeling results. Comparison of experimental data [12] and 1D modeling results for pressure history during the cooling and rewarming of the isochoric chamber illustrated in Fig 2A. Also overlaid are the equilibrium pressure values according to the phase diagram.

Fig 5 displays the measured isochoric chamber pressure as a function of the temperature measured on its outer surface. The thermal protocol for that experiment started with an initial equilibration step at 0°C. Next, the isochoric chamber was cooled in a step-like fashion from 0°C to -30°C in 5°C increments. Each of these temperature steps followed an unspecified cooling rate when the temperature of the alcohol solution cooling bath was set to a new set point. Based on evaluation of the experimental results, the time required to reach thermal equilibrium at each step was shorter than the 30 min allotted to it. Since the unique behavior of water expansion upon ice formation prevails only along the liquidus curve of phase Ih ice—between 0°C and -22°C [10]—the current benchmarking study excludes the lower experimental temperature steps of -25°C and -30°C. Similarly, the rewarming protocol progressed in a step-like fashion, in the same temperature increments and step durations, but in a reverse order.

Since the actual cooling rates and rewarming rates for the respective temperature steps, and also the heat transfer coefficient by convection within the cooling chamber are all unknown, the stepwise steady state conditions are primarily used for comparison in the current study, while the transient response is discussed qualitatively later. Nonetheless, a parametric estimation to fit experimental data with modeling results suggests average cooling rates and rewarming rates of 1°C/min and convective heat transfer coefficient of 900 W/m^2^ K, which were used for modeling in this study. It is quite conceivable that different combinations of temperature rates and heat transfer coefficients would yield closely related results, as discussed below. Nonetheless, this uncertainty does not affect the generality of the conclusions presented herein.

Fig 1 displays good agreement between steady state modeling results and theoretical values on the liquidus curve of the phase diagram, for the same temperature steps used in the benchmark experimental study [12], but for longer duration than experimentally practiced for reasons that are discussed below. Fig 5 displays the modeled temperature-pressure history at the center of the isochoric chamber, in comparison with experimental data (solid lines). Fig 5 also displays the modeling results for extended hold times at each temperature step (dashed lines). Based on the latter modeling results, the system is deemed approaching steady state after 120 min hold time, with the maximum pressure difference of 1.5% (0.92 MPa) between modeling and theoretical data, observed for the -5°C temperature step. At the lower temperature boundary for ice Ih formation, -22°C, the difference between theoretical and modeled values is only 0.92 MPa or 0.4% of full range (not shown on Figs 1 and 5).

When comparing the theoretical results with experimental data based on Fig 5, it should be emphasized that that the modeled temperature refers to the center of the domain, while the experimentally measured temperature refers to the outer surface of the isochoric chamber. Note that the absence of pressure elevation in the first step of cooling, at -5°C, is attributed by the authors of the experimental study to the phenomenon of supercooling. In broad terms, supercooling refers to the suppression of crystallization below the equilibrium phase transition temperature due to the absence of sufficient heterogeneous nucleation sites [39]. Overall, the maximum pressure difference during cooling between the proposed model and experimental results at the 30 min mark (the end of each experimental step) is 1.96% (3.67 MPa), observed for the temperature hold of -20°C. On the other hand, the maximum pressure difference during rewarming between the proposed model and experimental results at the 30 min mark is 13.5% (8.04 MPa) observed for the temperature hold of -5°C.

It can be concluded that theoretical results and experimental data display similar trends in Fig 5. However, while modeling steady state pressure levels converge to the phase diagram values, the experimental data show a somewhat increased deviation from the phase diagram values with the decreasing temperature. Experimental results also suggest approaching steady state condition sooner. For example, for cooling to -20°C, the difference between computed and the theoretical equilibrium pressure is 1.34% or 2.59 MPa at 30 min but only 0.42% or 0.82 MPa at 120 min. The reasons for the deviation from equilibrium theoretical values in the experimental data may be attributed to compliance of the isochoric chamber and internal parts of the pressure transducer, calibration of the pressure transducer, and/or that the isochoric chamber has not reached thermal equilibrium. However, the latter possibility seems less likely as the steady state pressure during the cooling steps and the respective rewarming steps is in a very close range. The compliance of the thick steel wall of the isochoric chamber may be negligible as addressed in the second part of the Discussion section, but the compliance of the internal parts of the pressure transducer and its fittings could potentially play a significant role in that effect.

The authors of this study cannot evaluate the experimental temperature-pressure behavior below -22°C based on the equilibrium phase diagram, since the material behavior from the literature is inconsistent with the measured data. Below -22°C, water does not exist in a liquid form at equilibrium. Going back to the fundamentals of mechanics, the stress is described by a nine-components tensor at every point in the domain, where six components describe shear stresses, while the remaining three components describe the normal stresses. In a stationary fluid the shear stress components are zero by definition, while the three normal stresses are identical, defining the concept of a hydrostatic pressure. In a solid however, all the nine stress components may be non-zero, and there is no condition requiring the normal stress components to be equal. Once all the water contained in the isochoric chamber solidifies, one may expect shear stresses to develop within the domain due to the constraints applied by the rigid walls. At this stage, the geometry of the isochoric chamber also starts to play a key role in the development of variable shear and normal stresses, and the concept of a hydrostatic pressure as being constant across the domain and in any direction vanishes.

Since the normal stresses may vary across the domain, once it has completely solidified, the experimental data obtained by the pressure transducer below -22°C should not be regarded as a pressure across the isochoric chamber anymore, but only as one of the three normal stresses and only at the point of measurement, assuming that the pressure transducer is capable of measuring that in a solid. It is for this reason that the concepts of pressure in stationary fluid are insufficient to interpret experimental data below the liquidus curve as suggested in [18], and the analysis of solid mechanics must take front and center stage to evaluate the relationship between the various stress tensor components.

Furthermore, while water expands upon solidification when forming ice Ih below 207.4 MPa, it contracts upon solidification when forming ice III above that pressure (pressures higher than the triple point on Fig 1). Continuing with a hypothetical thought experiment from this point onwards, even if the outcome of the solid mechanics analysis would suggest all shear stresses to be zero, it would still be difficult to explain why the experimental pressure measurements are now not reduced with the decreasing temperature. Furthermore, if solid-solid phase change is considered between ice Ih and ice III, the time scale for that to happen is unknown to the authors but could expected much longer than the experimentation time scale.

The discussion now turns to the comparison of experimental data with modeling results during the transient portion of the various temperature steps. A more detailed comparison of the temperature-pressure history suggests slower initial increase in pressure with the decreasing temperature in the experimental work, and faster convergence to a steady state value at the later stage of cooling, for each respective step. The same characteristics are displayed during rewarming, but in the reverse direction. For the modeling on the other hand, the parametric estimation for the heat transfer coefficient was performed such that the difference between the experimental and modeling histories is minimized during cooling, where the only variable is heat transfer coefficient. While that represents a practical approach to parametric estimation when many parameters of the experimental conditions are unknown, this study does not claim that the exact heat transfer behavior is modeled.

**Fig 6:**
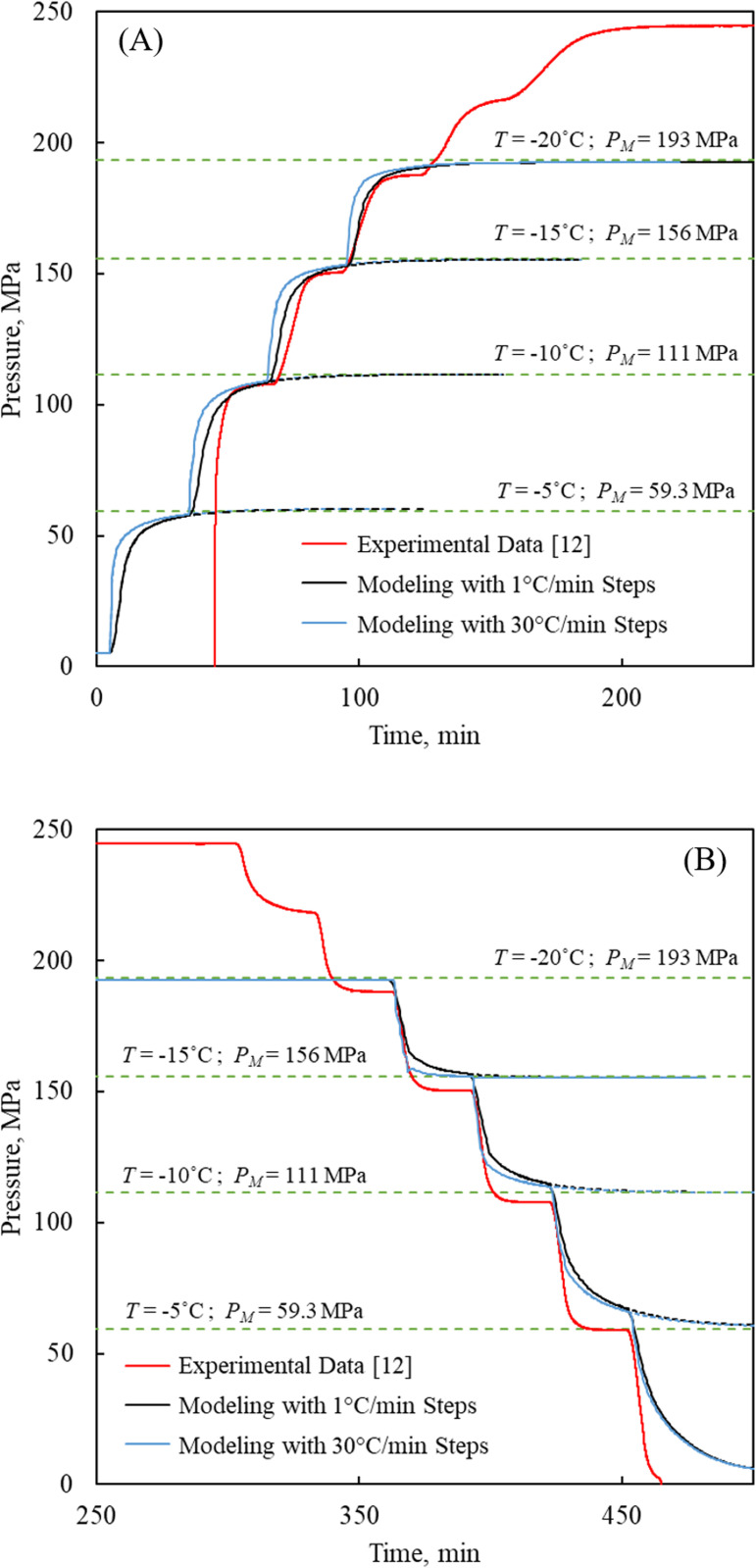
Temperature-pressure histories in the isochoric chamber illustrated in Fig 2A subject to various temperature rates. Temperature-pressure histories subject to (A) stepwise cooling of the isochoric chamber; and (B) stepwise rewarming.

Fig 6 displays what would happen if the cooling and rewarming rates are increased from 1°C/min to 30°C/min, resulting in a modest effect on the modeling results, an effect which cannot explain the different transient trends between experimentation and modeling. This leads the discussion to the limitation of modeling in the current comparison: (i) the mathematical model is 1D, while the experimental conditions create a 3D problem; (ii) the mathematical model assumes infinitely rigid walls (Eq (18)), while the isochoric chamber, the pressure transducer, and its fittings may display some level of mechanical compliance; (iii) the mathematical model presents the temperatures at the center of the domain, while the temperature measurements are taken on the outer surface of one of the bulky isochoric chamber couplings; and, (iv) the mathematical model does not take into account the kinetics of crystallization, which would only further slowdown the approach to steady state at any specific temperature. The limitation of the experimental setup on the other hand, is related to the fact that the temperature sensor on the outer surface of the domain may respond the fastest to changes in environmental conditions, while water at the center of the couplings (Fig 2) may be the slowest to respond to the same changes. Despite the lack of experimental data to allow for a full-blown 3D thermal analysis, the modeled pressure-temperature history resembles the experimentation measurements to a high degree. For these reasons, this section of discussions attributes higher certainty to the comparison at thermal equilibrium.

Finally, given the integration of solid mechanics into the proposed modeling, an additional 1D thermo-mechanics analysis was performed, where the material properties of the steel chamber (Type 316) were also considered for the experimental system. In this case, the steel is considered as linear elastic, where its properties are listed in Table 3. When the steel container is considered, the boundary condition defined in Eq (18) is relaxed and replaced by a continuation in displacement between the water and the solid wall on the inner surface, while zero radial stress on the outer surface of the container is imposed instead, Eq (19). The pressure history for this case, measured at the center of the water domain, is displayed in Fig 5. Results of this analysis show a total displacement of 1.59×10^−3^ mm on the water-wall interface when the isochoric chamber reaches -20°C, which represents 6.67×10^−3^% deformation. The later value is negligible in comparison with the 3% linear expansion of water in standard atmospheric pressure and in a stress-free setup (equal to 9% of stress-free volumetric expansion). This supports the modeling approximation that the steel chamber behaves isovolumetrically.

To further investigate the effect of the thick-walled container, four additional cases are considered with varying wall thicknesses in addition to the rigid boundary condition in Eq (18): 1.19 mm, 2.38 mm, 4.76 mm and 9.52 mm. The wall thickness in of the isochoric chamber used for experimentation is 4.76 mm and the other studied cases represent variation of ×¼, ×½, and ×2 from that original value. For modeling purposes, the rigid boundary condition analysis assumes zero wall thickness. For the comparison between these cases, the duration of each surroundings temperature step was maintained at 30 min, including an initial cooling at a rate of 20°C/min. Furthermore, a heat transfer coefficient of 350 W/m^2^K is considered to mimic the conditions inside a controlled-rate cooling chamber, such as the Kryo 10 [40].

**Fig 7:**
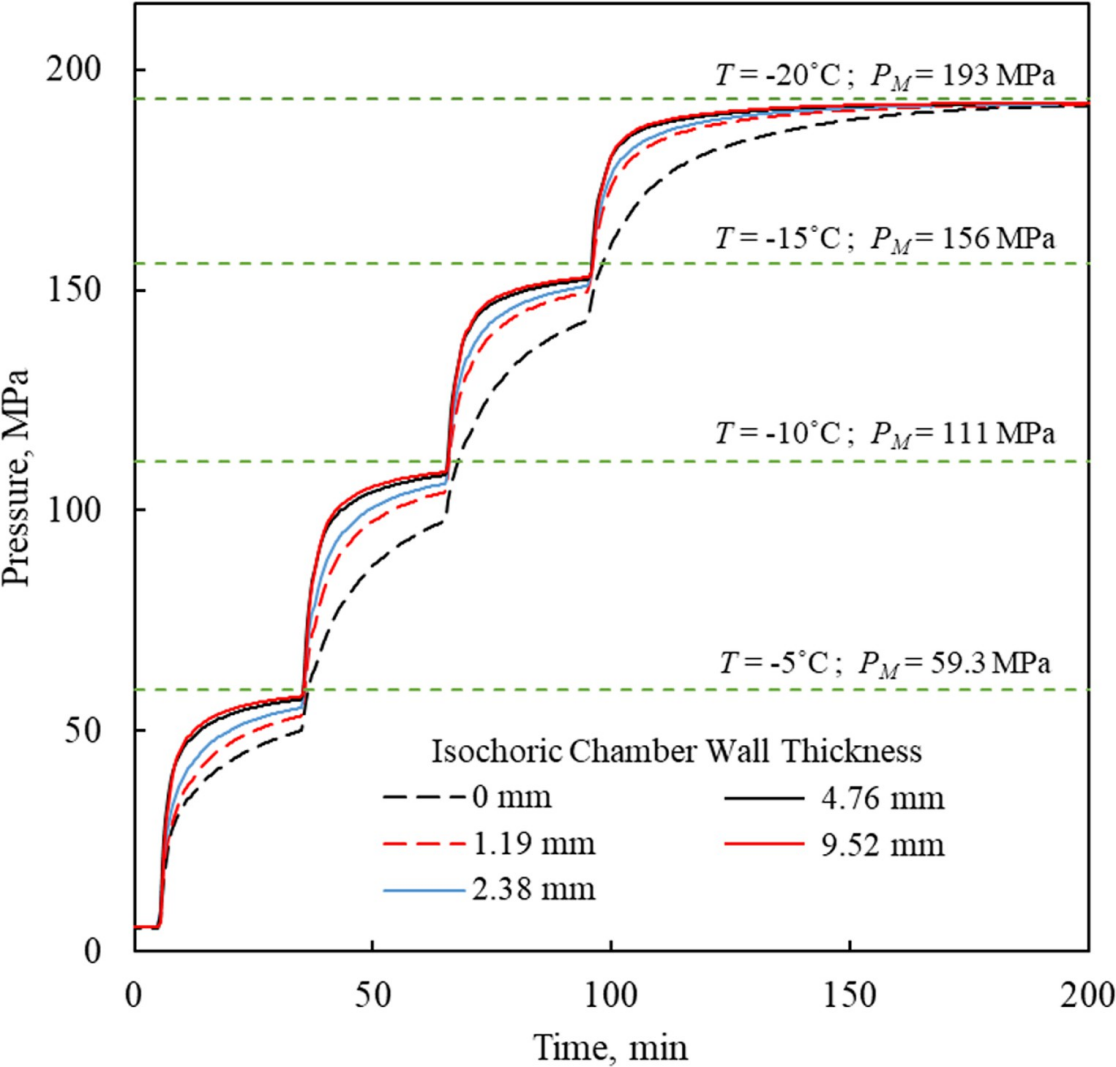
The effect of wall thickness on the temperature-pressure history during isochoric cooling. The analysis is 1D for a geometric model similar to the experimental setup, where the cooling rate between subsequent temperature steps is 20°C/min while the heat transfer coefficient on the outer surface of the system is 350 W/m^2^K.

The container wall thickness is not only related to the maximum stress in the container, but also influences the thermal performance of the system. The calculated pressure history at the center of the isochoric chamber for the various container wall thicknesses is displayed in Fig 7. The temperature response rate of the isochoric chamber to changes in its surroundings temperature increases with its increasing wall thickness, resulting in a shorter period required to reach steady state, Fig 7. This may be perceived counterintuitive at first glance, since the thermal mass of the system also increases with the increasing wall thickness and thereby tending to slowdown the thermal response. At the same time, the outer surface area of the container increases with the increasing container diameter, and thereby increasing the rate of heat exchange between the isochoric chamber and its surroundings. The effect of increasing heat exchange to the surroundings with the increase in the wall thickness of a cylinder is commonly associated with thermal insulators in the fundamental textbooks of heat transfer [41]. There, this effect is shown to be bounded by the ratio of the thermal conductivity in the wall to the heat transfer coefficient by convection to the surroundings, yielding the concept of the critical radius. If the actual outer radius of the isochoric chamber is smaller than the critical radius, the counterintuitive effect described above prevails, which is the case in the current reported case studies. It follows that not only the solid mechanics analysis is required for the design of isochoric containers to meet failure criteria, but also thermal analysis should be included in the design to improve its thermal performance, while the underlying principles of thermodynamics cannot shed light on either aspects of system performance.

### Scale-up isochoric cryopreservation

The discussion now turns to a 3D analysis of three idealized and geometrically similar isochoric chambers, Fig 2B, in volumes of 3 ml, 150 ml and 500 ml. The 3 ml chamber resembles the experimental chamber discussed above for reference, while the 500 ml volume represents a practical chamber size that would be used for the preservation of large organs or limbs as examples. In all cases, the initial temperature is 10°C and the cooling chamber is cooled at a constant rate of 0.4°C/min down to -10°C, after which the cooling chamber temperature remains constant. The same heat transfer coefficient from the above validation study is selected (900 W/m^2^ °C).

**Fig 8:**
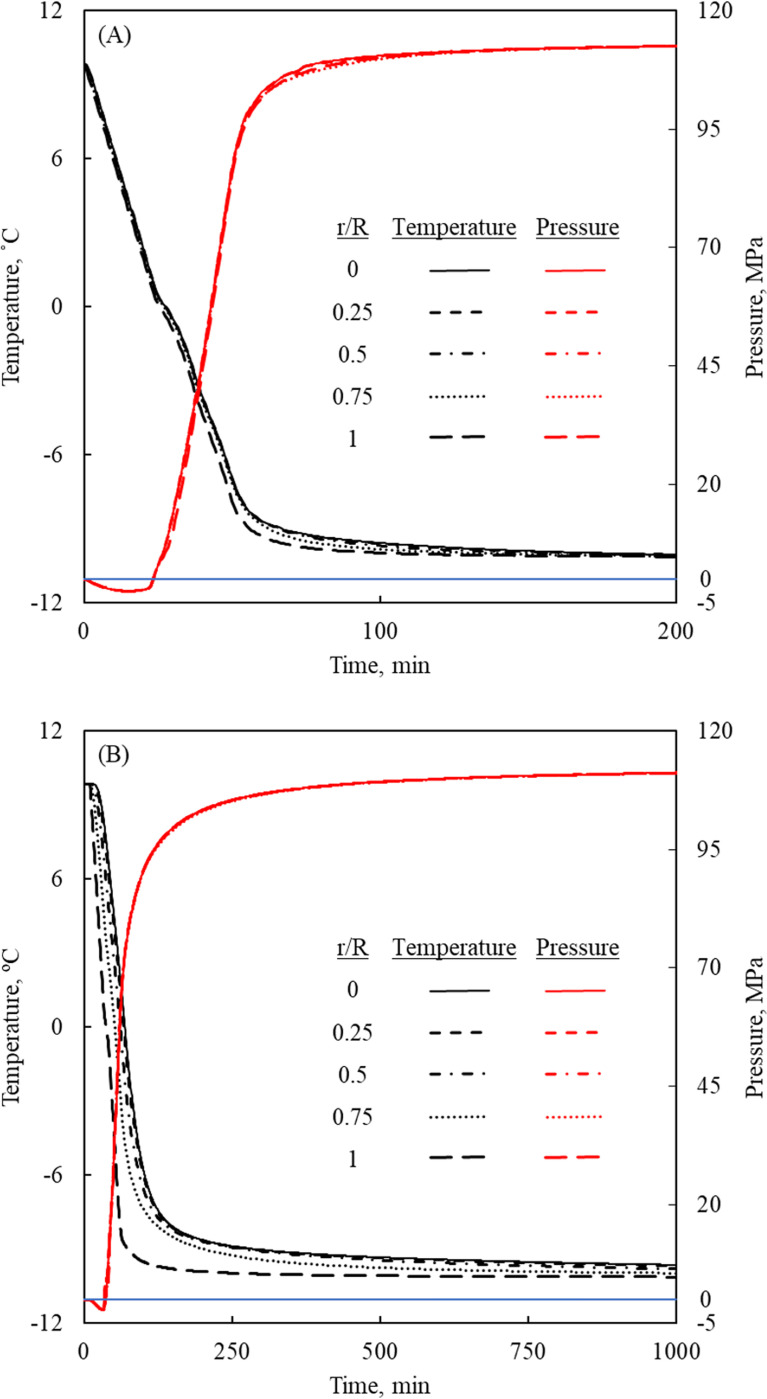
Temperature and pressure histories at five representative points in the domain shown in Fig 2B. The representative points are in the radial direction at mid-height of the domain for: (A) a 3 ml chamber having a diameter of 11.7 mm and height of 29.2 mm; and (B) a 500 ml chamber having a diameter of 63.5 mm and height of 158.7 mm.

Fig 8A displays the temperature and pressure histories at five representative radial locations at the mid-height of the 3 ml cylindrical chamber, Fig 2B. If can be seen from Fig 8A that the isochoric pressure decreases with the decreasing temperature between 10°C and 4°C, due to the initial contraction of water with the decreasing temperature. This pressure trend reverses below 4°C, but becomes significant only at the onset of ice formation as intended. The pressure decrease early on can get close to absolute zero pressure (i.e., vacuum), which can trigger nucleated evaporation [42]. The heat required for evaporation could slightly assist in further cooling the liquid water. However, since the proposed mathematical model does not include evaporation, and since the associated pressure variation is negligible (0.1% of full scale), this initial effect is not analyzed in the current study. Nonetheless, from a practical perspective, the resulting vapor bubbles can serve as ice nuclei during solidification under extreme conditions and when acknowledging that a significant temperature distribution may prevail across the isochoric chamber. The related extreme conditions are beyond the scope of the current study.

It can be seen from Fig 8A that water is cooled relatively uniformly across the isochoric chamber. The maximum radial temperature variation across the isochoric chamber is found to be 0.97 ˚C at 50 min from the beginning of cooling. It can be seen from Fig 8A that the pressure also elevates uniformly. As the isochoric chamber approaches equilibrium of -10°C after 200 min, the pressure elevates to 112.9 MPa, which is 1.5% higher than the equilibrium pressure of 111.2 MPa on the phase diagram. That pressure difference at steady state is associated with a numerical error due to FEA discretization and possible numerical errors in the coupled problem. The maximum instantaneous pressure difference across the chamber is found to be 3.66 MPa at *t* = 30.8 min (3.2% of full scale).

The small deviation from pressure uniformity across the isochoric chamber in the process described above could possibly be attributed to numerical errors in modeling. On the other hand, such deviation from pressure uniformity could also be attributed to the combined effects that: (i) some parts of the domain gradually turn into solid in the process of cooling; (ii) the pressure is defined as the average normal stresses at a point, Eq (17); and (iii) the normal stresses are not necessarily equal in a solid as discussed above, while some portion of the domain does turn into solid. However, since the entire process takes place along the liquidus curve of the phase diagram, the variation in normal stresses would be quite small. In practice, a single pressure transducer at the edge of the domain cannot pick up such variations across the domain, and definitely not in a solid portion of the domain. Therefore, whether this pressure variation is a real phenomenon or merely a modeling and/or numerical artifact remains an open question at this stage.

A significant temperature variation can be observed in the case of a larger isochoric chamber, Fig 8B, while the pressure variation across the domain remains almost uniform. Based on the thermal histories at the various locations, one may assume that ice forms on the walls of the isochoric chamber, which suggests that the cryopreserved material should be placed at the center of the domain to protect the biological cells from the hazardous effect of ice crystallization. The rate of ice formation at this stage of the process may be estimated using a freezing-front tracing numerical method [43].

During rewarming however, the pressure-dependent thermal history reverses and the outcome may be counterintuitive. For the purpose of discussion, assume a hypothetical rewarming process starting from an initial thermal equilibrium condition, which is the end state of the cooling process highlighted in Fig 8. At that stage, two regions may exist in the domain: liquid at the center of the domain surrounded by ice on the walls. From the same heat transfer considerations that the water in contact with the inner surface of the isochoric chamber cooled first during the cooling process, it is now rewarmed first during the beginning of recovery from cold storage. Now, ice that is in contact with the inner surface will melt first, resulting in possibly three regions in the isochoric chamber: colder liquid region at the center, warmer liquid region along the walls and an ice region in between. If the reduced pressure equilibrates between the two water regions, the water at the center may now tend to freeze since it would be too cold for the reduced pressure according to the liquidus curve on the phase diagram.

Practical reasons for pressure to equilibrate between the two liquid water regions may be associated with fractures within the ice region due to thermo-mechanical stress or merely due to a mechanical stress driven by a significant pressure difference, where the intermediate ice region acts in practice as a pressure vessel. Even more so, it is conceivable that a completely ice-encapsulated inner liquid region may never from due to thermal effects such as heat transfer through fittings, pressure transducer, etc. Regardless of the reason for pressure equilibration across the isochoric chamber during rewarming, when it does happen, the risk for ice crystallization at the center is imminent. Moreover, the surface of the cryopreserved material is at the liquidus curve conditions and the tendency of surface roughness to promote ice nucleation, and the risk for ice crystallization in the cryopreserved material or on its surface only increases. Of course, the current study does not account for the kinetics of crystallization and hence the location of ice formation is based on thermal considerations only. In this broader context however, the current discussion aims at: (i) drawing the attention to the fact that rewarming is not the inverse of cooling in the sense that more phase regions may coexist during rewarming, and (ii) alerting about conditions for which ice crystallization may develop at the center of the domain during rewarming. The effect of such ice crystallization remains undetermined, while acknowledging literature experimental evidence on good isochoric cryopreservation outcome.

With the above considerations in mind and while ignoring the actual distribution of ice in the isochoric chamber, the relative portion of ice by mass within at steady state can be calculated by [3]:

$$F_{ice}=\frac{\rho_{l}^{-1}(T_{m})-\rho_{chamber}^{-1}}{\rho_{l}^{-1}(T_{m})-\rho_{s}^{-1}(T_{m})}$$ (27)

where the inverse of density is also defined as specific volume, and the overall density of the isochoric chamber, *ρ*_*chamber*_, is the total mass of water in the isochoric chamber divided by its volume. *ρ*_*chamber*_ remains constant when the displacement at the inner surface of the isochoric chamber can be neglected, or otherwise calculated from the mechanics solution when taking into account the deformations in the isochoric chamber walls.

The amount of ice calculated for the stepwise cooling displayed in Fig 5 is 24.3% at -5°C, 38.1% at -10°C, 47.2% at -15%, 53.6% at -20°C, and 55.9% when approaching -22°C while increasing the pressure during cooling. Recall, that liquid water cannot exist below -22°C unless supercooled, a condition which is less likely to happen in any of the cases discussed in this study. Modeling the effects of water cooling and ice formation in the vicinity of the triple point remains an unresolved challenge.

## Summary and conclusions

A new model for the thermo-mechanics problem associated with isochoric cryopreservation is presented in this study. This model is unique in the sense that it integrates concepts from the disparate fields of thermodynamics, heat transfer, fluid mechanics, and solid mechanics. The novelty in this study is in treating the cryopreserved material as having a pseudo-viscoelastic behavior over a very narrow temperature range, without affecting the mechanics behavior of the material in the rest of the domain. This unique approach permits treating the domain as a continuum, while avoiding the need to trace freezing fronts and sperate the analysis to liquid and solid subdomains. Consistent with the continuum approach, the heat transfer problem is solved using the enthalpy approach. The thermodynamics aspects of isochoric preservation are introduced through physical properties, and the temperature-pressure dependency of melting conditions through the phase diagram. Furthermore, the underlying model approach is designed to simultaneously solve the thermo-mechanics problem in the medium undergoing isochoric preservation and its container.

The application of a pseudo-viscoelastic material model is inspired by continuous efforts to analyze thermo-mechanics effects during cryopreservation by vitrification subject to isobaric boundary conditions. There, the vitrifying material behaves as fluid in higher temperatures, and as a linear-elastic material below glass transition in any practical time scale—just like ice in the solid state. The proposed material model assumes a similar behavior, but over a dramatically narrower temperature range. This approach facilitates treating the material as a continuum, without the need to trace a freezing front in order to distinguish solid from liquid. It is further argued in this study that the application of a freezing front tracing method could be impractical due to the co-existence of multiple freezing fronts during rewarming, and furthermore due to the geometric complexity introduced in 3D practical isochoric chamber shapes.

Detailed analysis in this study is limited to isochoric cooling of pure water between standard atmospheric conditions and the triple point of liquid water, ice Ih, and ice III (-22°C and 207.4 MPa), due to the availability of literature data on physical properties. Nonetheless, this modeling approach is applicable to isochoric cooling of water in any temperature and pressure range with no modifications, pending the availability of physical properties. Furthermore, the proposed model is also applicable to isochoric vitrification, by substituting the pseudo-viscoelastic material model with the real viscous model of the vitrifying material, as has been demonstrated for vitrification under isobaric boundary conditions previously [23–26].

Results of this study display good agreement with phase-diagram data at steady state, where the liquidus curve can be recreated to a high certainty. Results of this study also display good agreement with experimental data obtained previously by Ukpai at al. [18], which provided a venue to bridge the gap between limited pressure and temperature measurements and the continuum, while discussing experimentation aspects of isochoric cryopreservation. The proposed model is further demonstrated on a 3D and axisymmetric problem of isochoric cooling of water, while discussing scale considerations and transient effects. Finally, the limitations of the proposed model are critically discussed, while drawing attention to the counterintuitive possibility of ice crystallization at the center of the domain during recovery from cryogenic storage. Arguably, this study presents the most advanced thermo-mechanics model to solve practical problems relating to isochoric cryopreservation.
